# Exposure to psychosocial risk factors in the context of work: a systematic review

**DOI:** 10.1590/S1518-8787.2016050006129

**Published:** 2016-05-06

**Authors:** Cláudia Fernandes, Anabela Pereira

**Affiliations:** I Centro de Apoio Tecnológico à Indústria Metalomecânica. Porto, Portugal; II Departamento de Educação e Psicologia. Universidade de Aveiro. Aveiro, Portugal

**Keywords:** Psychosocial Impact, Occupational Risks, Working Environment, Professional Autonomy, Job Satisfaction, Mental Health, Occupational Health

## Abstract

**OBJECTIVE:**

To analyze the scientific literature about the effects of exposure to psychosocial risk factors in work contexts.

**METHODS:**

A systematic review was performed using the terms “psychosocial factors” AND “COPSOQ” in the databases PubMed, Medline, and Scopus. The period analyzed was from January 1, 2004 to June 30, 2012. We have included articles that used the Copenhagen Psychosocial Questionnaire (COPSOQ) as a measuring instrument of the psychosocial factors and the presentation of quantitative or qualitative results. German articles, psychometric studies or studies that did not analyze individual or work factors were excluded.

**RESULTS:**

We included 22 articles in the analysis. Individual factors, such as gender, age, and socioeconomic status, were analyzed along with work-related factors such as labor demands, work organization and content, social relationships and leadership, work-individual interface, workplace values, justice and respect, personality, health and well-being, and offensive behaviors. We analyzed the sample type and the applied experimental designs. Some population groups, such as young people and migrants, are more vulnerable. The deteriorated working psychosocial environment is associated with physical health indicators and weak mental health. This environment is also a risk factor for the development of moderate to severe clinical conditions, predicting absenteeism or intention of leaving the job.

**CONCLUSIONS:**

The literature shows the contribution of exposure to psychosocial risk factors in work environments and their impact on mental health and well-being of workers. It allows the design of practical interventions in the work context to be based on scientific evidences. Investigations in specific populations, such as industry, and studies with more robust designs are lacking.

## RESUMO

**OBJECTIVO:**

Analisar a literatura científica acerca dos efeitos da exposição a fatores de risco psicossocial em contextos de trabalho.

**MÉTODOS:**

Realizou-se revisão sistemática usando os termos “psychosocial factors” AND “COPSOQ” nas bases de dados PubMed, Medline e Scopus. Foi analisado o período entre 1^o^ de janeiro de 2004 e 30 de junho de 2012. Foram incluídos artigos que utilizaram o *Copenhagen Psychosocial Questionnaire* (COPSOQ) como instrumento de medida dos fatores psicossociais e a apresentação de resultados quantitativos ou qualitativos. Foram excluídos artigos em alemão, estudos psicométricos ou estudos que não analisavam fatores individuais ou do trabalho.

**RESULTADOS:**

Foram incluídos 22 artigos na análise. Fatores individuais como género, idade e estatuto socioeconómico foram analisados a par com fatores relacionados com o trabalho como exigências laborais, organização do trabalho e conteúdo, relações sociais e liderança, interface trabalho-indivíduo, valores no local de trabalho, justiça e respeito, personalidade, saúde e bem-estar, comportamentos ofensivos. Foi analisado o tipo de amostra e os designs experimentais aplicados. Existem públicos mais vulneráveis como os jovens e os migrantes. O ambiente psicossocial de trabalho deteriorado associa-se a indicadores de saúde física e saúde mental débeis. É também fator de risco para o desenvolvimento de quadros clínicos de gravidade moderada a severa, predizendo absentismo ou intenção de deixar o trabalho.

**CONCLUSÕES:**

A literatura fundamenta o contributo da exposição a fatores de risco psicossocial em ambientes de trabalho e o seu impacto na saúde mental e no bem-estar dos trabalhadores. Permite que o desenho de intervenções práticas em contexto de trabalho tenha como base evidências científicas. Faltam investigações em populações específicas como a indústria e estudos com delineamentos mais robustos.

## INTRODUCTION

The exposure to psychosocial risks in work contexts is one of the biggest challenges to occupational safety and health. This is due to the continuous change and evolution of nature and work organization^a^ ^,^ ^b^ ^,^ ^c^ and to their impact on people, organizations, societies^14^, and policies. The environment and nature of work influence the general health and welfare of the human being^17^ ^,^ ^22^. The International Labour Association defines psychosocial risk in terms of interaction between work content, work organization and management, other organizational and environmental conditions, and the skills and needs of the worker^d^. These interactions proved that there are risks to the health of workers and differences in how they experience them. Literature shows a consensus about the nature and identification of psychosocial risks^16^ ^,^ ^17^, such as labor demands, work organization and content, social relationships and leadership, work-individual interface, workplace values, justice and respect, health and well-being, and offensive behaviors. New or renewed forms of work, social contexts of interaction, demographic changes, migration flows, economic crisis on a global scale, new technologies, renewed business models, and management of business/logistics networks cause the appearance of new or different risks, which often take the form of emerging or unknown risks. These risks might not be represented in scientific publications^6^ because of the operational nature of the measurement of exposure to psychosocial risks in work context, but which may have a major impact at different levels.

The study on exposure to psychosocial factors and stress are interrelated^17^. To this end, many admeasurement tools and techniques are used in both constructs. Some of the most popular and used instruments are the Job Content Questionnaire (JCQ)^10^, the General Nordic Questionnaire for Psychosocial and Social Factors at Work (QPS)^19^, the Effort-Reward Imbalance Questionnaire (ERI)^33^, and the Copenhagen Psychosocial Questionnaire (COPSOQ)^14^. COPSOQ shows differentiating features from most of these instruments, in particular the JCQ and ERI, because it is not based only in a theoretical model explaining the relationship between psychosocial risks, work environment, and health^5^, but in a systemic approach. Because of its practicality, COPSOQ can include more relevant dimensions to the investigation, which might not be covered by previously validated models. It can an also include less studied factors. We chose studies that included this instrument because, besides its multifaceted approach^e^, (i) it has at its base the epidemiological method, which defines units of analysis in three sections (improve, maintain, and promote) and allows a practical valuation measure and workstation intervention; (ii) it incorporates reference values for different countries, sectors, and particular occupations, allowing the definition of threshold levels for exposure to psychosocial risks (like the analysis of other types of risks, e.g., chemical contaminants); (iii) it is applicable to all types of companies/institutions, since it had in its genesis the analysis and prevention of occupational risks.

No systematic review of the literature about exposure to psychosocial risks in work contexts focusing on individual and work factors is reported. Besides, the systematic studies on instruments that assess these psychosocial factors are restricted. This study aimed to analyze the scientific literature about the effects of exposure to psychosocial risk factors in work contexts.

## METHODS

Systematic review of the literature. The criteria of the Preferred reporting items for systematic reviews and meta-analyses (PRISMA)^f^ were applied. We identified scientific articles published in international journals using a systematic search in the databases in digital format: PubMed, Medline; in a second stage, at the Scopus database. We considered the studies published from January 1, 2004 to June 30, 2012. The research equation was [“PSYCHOSOCIAL FACTORS”], and refined to [“PSYCHOSOCIAL FACTORS” AND “COPSOQ”] because of the high number of correspondences. The study was restricted to articles published in English. The research using the full equation returned 79 abstracts with 30 correspondences in both databases (PubMed and Medline), resulting in 27 articles in full text. Of these 27, four psychometric studies and one study that did not incorporate individual or work factors were excluded, resulting in 22 articles. At the second stage of the research, on Scopus database, 13 articles were excluded because they were duplicated. The 22 articles in full text resulting from the structured research of the literature were analyzed in the review process of the literature. Cataloging and identification of repeated references were made by the software program of bibliographical referencing EndNote (Table 1; Figure).

**Table 1:** Criteria for inclusion and exclusion of studies about exposure to psychosocial risk factors in work contexts.

Criteria	

Inclusion	Exclusion
Use of COPSOQ for measuring psychosocial risks Presentation/analysis of quantitative/qualitative data about individual or work-related factors Studies published between January 1, 2004 and June 30, 2012.	No use of COPSOQ for measuring psychosocial risks No presentation/analysis of quantitative/qualitative data about individual or work-related factors Outside the research period Psychometric study^a^ Repeated Another language^b^

COPSOQ: Copenhagen Psychosocial Questionnaire

^a^ Psychometric validation studies of scales/instruments.

^b^ Original documents in other languages besides English.

**Figure:**
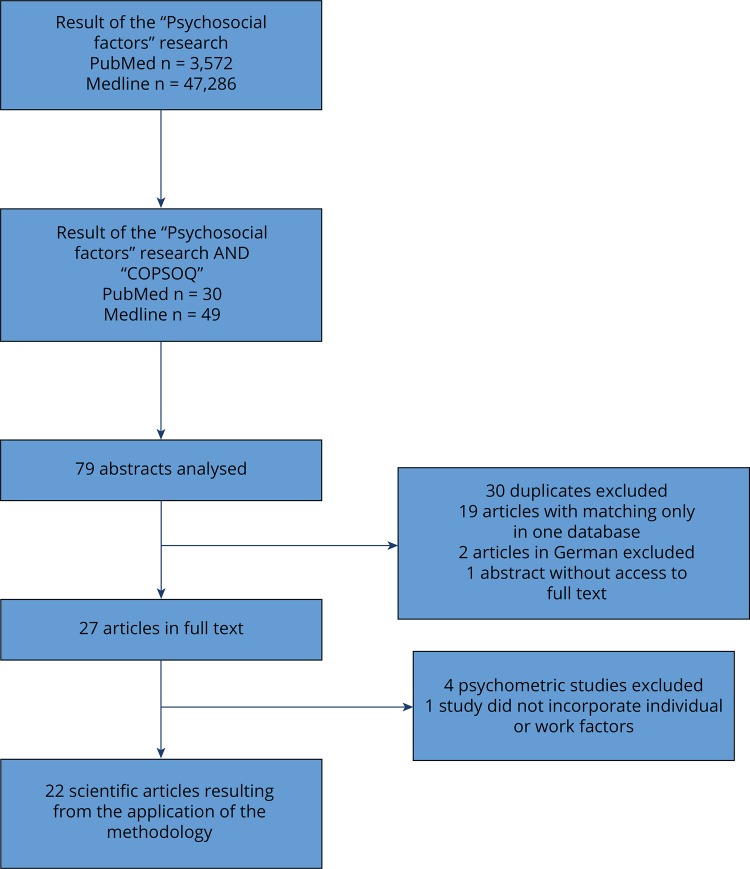
Flowchart – application of the inclusion and exclusion criteria to the researched studies.

Exposure to work-related psychosocial factors was codified according to the categories of COPSOQ: labor requirements, work organization and content, social relations and leadership, work-home interface, workplace values, personality, health and wellness, and offensive behaviors^13^. The individual factors (age, gender) were also coded.

Information on the type of study, sample, country, activity sector, main findings, and conclusions was collected, compiled and analyzed.

## RESULTS ANALYSIS AND DISCUSSION

We included 22 articles according to the inclusion and exclusion criteria (Table 1). We covered 51,894 people, of nine different nationalities. In three studies, the nationality variable was not described. The most represented nationalities were Danish, mentioned in nine of the 22 studies, Swedish and Dutch, both present in four studies, followed by workers from Germany and Spain in three studies. Switzerland’s workers were present in two studies and those of Poland and China, in one. The working population in general (irrespective of sector or activity area) prevailed in the study (48.0%), followed by health care workers (33.0%), and the staff of prison services (14.0% of people covered in all the analyzed studies); civil servants, hoteliers, musicians, services, and farmers were also studied. It was used the correlational study methodology the most, and the longitudinal methodology was in the basis of the study, covering approximately 0.7% of all people studied. Four studies used methodology combined with the longitudinal design (average duration of 13 months), increasing the robustness of the research and of the obtained results. The most studied categories in the study of exposure to psychosocial factors in the work place were: labor demands (18 studies), organization of work and content (14), social relationships and leadership and health and well-being (both 13 categories), work-individual interface (12), offensive behavior (eight), and the categories values in the workplace, justice and respect, and personality (seven). Eight studies used a global index of exposure to psychosocial risk factors (Table 2).

**Table 2:** Summary of information about the 22 studies included regarding exposure to psychosocial risk factors in work contexts.

	Author/Publishing year	Sample/Activity sector	Country	Methodology of the study	Main results	Individual factors and work-related factors ** Studied dimensions psychosocial factors Scale [subscale]
1	Albertsen et al.^1^ (2010)	n = 349 knowledge industry workers (i.e., employees of communication or knowledge exchange)	Denmark	Correlational Longitudinal (12 months)	Positive correlations were observed between the baseline of the quantitative requirements, conflicts and lack of role clarity, lack of recognition and cognitive stress symptoms. Influence and social support by the management are negatively associated with cognitive stress symptoms. Social support from colleagues did not present any significant relationship.	Self-esteem [cognitive stress] ** Labor demands [quantitative demands] Social relationships and leadership [transparency of labor role, labor role conflict, rewards/recognition, social support from colleagues] Health and well-being [cognitive stress]
2	Aust et al.^2^ (2010)	n = 399 hospital workers (nurses, midwives, assistants, laboratory technicians, others)	Denmark	Controlled intervention in workplace Longitudinal study (16 months)	The psychosocial environment deteriorated after the intervention, especially in the scales related to interpersonal relationship at work and leadership. There was no decrease in the scales that assessed the labor demands. The negative effects found in the scales of COPSOQ are partly because of the disappointment of workers with unrealized expectations.	Expectations ** Social relationships at work and leadership Labor demands
3	Burr et al.^5^ (2010)	n = 3,552 active workers	Denmark	Correlational	The COPSOQ scales had more positive results than those of ERI and of models of tension at work. Mental health deteriorated with more emotional demands and improved with more meaningful work. Regarding mental health, the new psychosocial risk factors have the potential to increase the predictive value of previous models (e.g., ERI).	Vitality, mental health ** Global index of exposure to risk factors Labor demands [emotional demands, demands to hide emotions] Work organization and content [meaning of work] Social relationships at work and leadership [predictability, quality of leadership] Work-individual interface Workplace values Personality Health and well-being Offensive behavior
4	Fuss et al.^7^ (2008)	n = 296 hospital doctors	Germany	Correlational	The interference of work in family was prevalent as part of the work-family conflict (when compared the sample with the population of the studied country in general). No significant gender differences were found. The work-family conflict was significantly correlated with burnout and with physical and cognitive symptoms of stress. Low levels of work-family conflict were correlated with greater job satisfaction, better perception of health in general, greater capacity for work, and higher levels of satisfaction with life. Organization and factors of interpersonal relationship at work were identified as significant predictors of work-family conflict. The function being studied showed differences for the country’s population in general, especially higher levels of stress and quality of life and lower levels of well-being in general.	Physical health Mental health Burnout Satisfaction with life Ability to work Gender ** Work-individual interface [work/family conflict; family/work conflict] Work organization and content Social relationships and leadership [social support from colleagues, social support from superiors]
5	Ghaddar et al.^8^ (2011)	n = 7,429 prison workers	Spain	Correlational	Quantitative and emotional demands, work-home conflict, low influence at work, low autonomy, low social support from colleagues, and stress had statistically significant negative associations with the ability to work. The association between age and ability to work was measured by work experience.	Ability to work, work experience Age ** Labor demands [quantitative demands, emotional demands] Work organization and content [influence at work] Social relationships and leadership [social support from colleagues] Work-individual interface [work-home conflict] Health and well-being [stress]
6	Holst et al.^9^ (2012)	n = 342 musicians of symphony orchestras	Denmark	Correlational	Women reported greater labor demands and higher levels of stress symptoms than men. Individuals in a given function are more susceptible to risk because of the pace, content and organization of work. The increase in labor demands, work organization and content, interpersonal relationships, leadership and work-home interface were significantly correlated with the increase in symptoms of stress.	Physiological stress Sex Function ** Labor demands Work organization and content Health and well-being [stress]
7	Kiss et al.^11^ (2012)	n = 990 public sector workers	Not available	Correlational	Quantitative demands, job insecurity, demands for hiding emotions, emotional demands, and commitment to the workplace were significantly associated with lower levels of recovery after work.	Recovery after work ** Labor demands [quantitative demands, emotional demands, demands to hide emotions] Work organization and content [commitment to the workplace] Work-individual interface [job insecurity]
8	Kolstrup et al.^12^ (2008)	n = 67 farmers in the dairy area and pig raising	Sweden	Correlational	No significant relationships were found between the psychosocial work environment and the development of musculoskeletal injuries. The probability of the physical work environment leading to musculoskeletal injuries is higher than the probability associated with the psychosocial work environment.	Musculoskeletal injuries ** Global index of exposure to risk factors [labor demands, work organization and content, social relationships and leadership, work-individual interface, workplace values, personality, health and well-being, offensive behaviors]
9	Kristensen et al.^14^ (2010)	n = 2,331 hospital workers	Denmark	Correlational Longitudinal (12 months)	Absences from work by illness increase with the decrease in the socioeconomic status (few exceptions). The social gradient in the absence from work by illness presented different durations and patterns.	Perception of overall health Absence from work by illness Function Socioeconomic status
10	Larsman et al.^15^ (2006)	n = 148 female workers that use computer and are 45 years old or older	Denmark, Netherlands, Sweden and Switzerland	Correlational	The labor demands influence the musculoskeletal injuries because of their mediating effect on stress. The effect of labor demands can be attributed to stress mechanisms.	Stress, musculoskeletal injuries Age ** Labor demands [quantitative demands, pace of work] Health and well-being [stress]
11	Li et al.^18^ (2010)	n = 3,088 nursing female workers	China	Correlational Longitudinal (12 months)	Increased emotional demands, decrease in work meaning, decrease in commitment to the workplace, and decrease in satisfaction were associated with the intention of leaving the job permanently. An unfavorable psychosocial work environment predicts the intention of leaving the job permanently.	Intention of leaving the job permanently Sex ** Work demands [emotional demands] Work organization and content [meaning of work, commitment to the workplace] Work-individual interface [work satisfaction]
12	Llorens et al.^20^ (2010)	n = 7,612 active workers with salary	Spain	Correlational	Exposure to psychosocial risks was associated with management practices. Exposure to good psychosocial environment was associated with participatory working methods, permanent employment contracts, not having the perception of being easily replaced, having superiors little authoritarian and little aggressive, not being inconsiderately treated, possibility of promotion, being paid according to the hours worked and function, working between 31 and 40 hours a week, and working regular shifts.	Management practices ** Global index of exposure to risk factors [labor demands, work organization and content, social relationships and leadership, work-individual interface, workplace values, personality, health and well-being, offensive behaviors]
13	Mache et al.^21^ (2009)	n = 203 doctors	Germany	Correlational	The perceptions of working conditions differ significantly depending on the ownership of the work. Labor satisfaction does not vary among different types of ownership of the work. The work demands and the available resources are associated with work satisfaction. The type of ownership of the work is not associated with work satisfaction.	Work satisfaction, ownership of the work ** Labor demands Work organization and content [influence at work] Work-individual interface [work satisfaction]
14	Moncada et al.^23^ (2010)	n = 10,044, in which: Danish workers (3,359), Spanish workers (6,685)	Spain, Denmark	Correlational	A relationship between socioeconomic status and work psychosocial environment was observed. Strong correlations between socioeconomic status and home country.	Socioeconomic status ** Global index of exposure to risk factors [labor demands, work organization and content, social relationships and leadership, work-individual interface, workplace values, personality, health and well-being, offensive behaviors]
15	Nielsen et al.^24^ (2009)	n = 1,737, in which: nursing staff (791), assistants (410), cleaning staff (255), dairy workers (281)	Not available	Correlational	Specific model for analyzed group. There is an association between psychosocial factors and low physical health (back pain). Differentiation between typically female occupations in replacement for women working conditions.	Physical health complaints, mental health complaints, behavioral stress, back pain, groups, sex ** Global index of exposure to risk factors [labor demands, work organization and content, social relationships and leadership, work-individual interface, workplace values, personality, health and well-being, offensive behaviors]
16	Nübling et al.^25^ (2010)	n = 889 geriatrics technicians – home work (412), geriatric nursing staff (313), other geriatric workers (164)	Germany	Correlational	The degree of negativity in the evaluation of psychosocial factors associated with the work demand were directly related to the amount of hours worked per week and the number of calls within period of prevention, i.e., during the “period of prevention”.	Working hours, “To be called”, “period of prevention”^a^ ** Labor demands
17	Nyberg et al.^26^ (2011)	n = 554 hotelier staff	Sweden, Poland, Italy	Correlational	Significant positive correlations were observed between destructive leadership at organizational level and weak psychosocial well-being at individual level.	Leadership, psychosocial well-being ** Social relationships and leadership [quality of leadership] Health and well-being
18	Olesen et al.^28^ (2012)	n = 285, in which: migrant workers (137), Dutch workers (148)	Netherlands	Correlational	Low prevalence of hypertension in the resident population. Significant association between meaning of work and hypertension, though with different standards for residents and migrants.	Blood pressure, migration ** Work organization and content [meaning of work]
19	Rugulis et al.^29^ (2010)	n = 3,188 active workers with salary	Netherlands	Correlational	The psychosocial environment predicts the absence from work for medically prescribed disease.	Sick leave ** Global index of exposure to risk factors [labor demands, work organization and content, social relationships and leadership, work-individual interface, workplace values, personality, health and well-being, offensive behaviors]
20	Sandsjö et al.^30^ (2006)	n = 146 female workers that use computer and are 45 years old or older	Denmark, Netherlands, Sweden, and Switzerland	Correlational	Although there are differences between groups regarding working conditions, such differences had no scale to constitute standards concerning musculoskeletal injuries.	Musculoskeletal injuries Age ** Global index of exposure to risk factors [labor demands, work organization and content, social relationships and leadership, work-individual interface, workplace values, personality, health and well-being, offensive behaviors]
21	Schenk et al.^31^ (2007)	n = 111 administrative staff and nursing staff	Not available	Correlational	There is no evidence that different mechanisms lead to low back pain in both analyzed professions. General recommendations regarding prevention and treatment of back injury for the professions under study were not found.	Musculoskeletal injuries (low back pain), profession ** Global index of exposure to risk factors [labor demands, work organization and content, social relationships and leadership, work-individual interface, workplace values, personality, health and well-being, offensive behaviors]
22	Sharipova et al.^32^ (2010)	n = 8,134 health care workers	Denmark	Correlational	No significant differences were found in the exposure to physical violence at the workplace by gender. Younger employees have higher risk of exposure to physical violence related to work. Workers exposed to high emotional demands at work showed a risk increased in more than twice regarding physical violence. Different components of the workplace seem to have an influence on the risk of exposure to violence related to work – e.g., type of institution. Lower rates of violence were found in workplaces with good quality of leadership, without role conflicts, and with high levels of commitment to the workplace.	Type of institution Gender, age ** Work demands [emotional demands] Work organization and content [commitment to the workplace] Social relationships and leadership [work roles conflicts, quality of leadership] Offensive behavior

COPSOQ: Copenhagen Psycosocial Questionaire; ERI: Effort-Reward Imbanlance Questionnaire

** the divider separates the “individual factors and work-related factors” from the “Studied dimensions related to psychosocial factors”. The scale and [the subscale(s)] are identified within the studied dimensions related to the psychosocial factors.

^a^ “To be called” or “period of prevention” – This period begins immediately after the end of the last normal working period and ends immediately before the beginning of the first normal period of subsequent work. The call to work is responsibility of the immediate superior or whoever replaces him/her, and must be restricted to interventions necessary for the operation of the installation or imposed by situations that affect the company’s economy and that cannot wait for assistance during the normal period of work. The worker shall register the verified abnormality, as well as the actions taken for its resolution and obtained results, about which the hierarchy immediately pronounces. Taken from Gabinete Estratégico e Estudos. Boletim do Trabalho e Emprego Digital, n. 37, vol 80, de 8 de Outubro de 2013 [Access on April 14, 2015]. Available from: http://bte.gep.msess.gov.pt/completos/2013/bte37_2013.pdf

In general, mental health and psychosocial well-being of employees have deteriorated regardless of the psychosocial factor evaluated, with special focus on the emotional demands^1^ ^,^ ^2^ ^,^ ^4^ ^,^ ^8^ ^,^ ^9^ ^,^ ^12^ ^,^ ^15^ ^,^ ^18^ ^,^ ^20^ ^,^ ^21^ ^,^ ^23^ ^-^ ^25^ ^,^ ^29^ ^-^ ^32^. This deterioration manifests itself in increased levels of physiological stress and cognitive stress^1^ ^,^ ^9^ ^,^ ^11^ ^,^ ^15^ on workers. This increased stress makes the workers express their answers: i) physiologically, with neuroendocrine and immune reactions; ii) emotionally, with manifestation of feelings of anxiety, depression or depressive symptoms, alienation, apathy, among others; iii) cognitively, with restriction of perception, alteration of concentration capacity and creativity, difficulty in decision-making; iv) behaviorally, e.g., with substance abuse – alcohol, tobacco, drugs, violence.

These symptoms are usually seen as resulting from stress and are associated with clinical conditions with inconstant intensity, frequency, and duration^g^, e.g., back pain, shoulder pain, headaches, or more complex conditions and with greater severity such as gastrointestinal disorders, ischemic heart disease, type II diabetes, mental illness and suicides associated with the workstation^h^ ^,^ ^i^. The worker perception of a deteriorated psychosocial environment appears associated with higher levels of absenteeism in the workplace, because of sick leaves, for instance.

Bad management practices and destructive leadership on the part of the managers showed harmful effects on the psychosocial organizational environment and on the individual well-being of workers^20^ ^,^ ^26^, such as: timetables above 40 hours per week, shift work, overtime unpaid, working methods not reported, little autonomy, impossibility of career development, excessive workloads. The number of hours worked per week influenced the way workers perceived the work environment in general. These two variables were negatively correlated^25^. Extended working hours, even if paid, as the hours worked in period of “prevention”, negatively influenced how the employees evaluated the psychosocial factors associated with the daily laboring. The good management practices related to effective management of working time and the stability of timetables and working contracts, associated with the cordiality and non-aggressive behavior by managers were favorable to the development and perception by workers of healthier psychosocial environments.

The relationship between work psychosocial environment and the prevalence of musculoskeletal injuries (MI) varied according to the population under study and the type of function performed^12^ ^,^ ^15^ ^,^ ^30^ ^,^ ^31^. With increasing age, also increases the likelihood of MI occurrence. Demanding work environments have led to a higher incidence of MI in blue-collar professions, because of its mediating effect on stress mechanisms. Labor demands, in particular the quantitative ones and imposed work rate presented themselves as factors relevant to the development of MI in the areas of the neck, shoulders, and lower back. These types of injuries also correlated with repetitive functions or in which the employee remained too long in the same position, constantly requesting the same muscle groups or functions that would require lifts and frequent manipulations. Worker complaints about the prevalence of MI were corroborated by medical examinations or medical diagnosis.

The interference of work on family life was an important factor for understanding the work-home conflict. The work and family demands have increased the need for effective management of time by the worker, and may lead to time conflict and impossibility to reconcile these demands. This interference was more pronounced in females. This factor presented itself as an increased risk in the event of bad interpersonal relationships with coworkers and lack of organization^5^ ^,^ ^7^ ^,^ ^8^ ^,^ ^11^ ^,^ ^12^ ^,^ ^18^ ^,^ ^20^ ^,^ ^21^ ^,^ ^23^ ^,^ ^24^ ^,^ ^29^ ^-^ ^31^. We considered the free time as period of rest and replacement of physical and mental energies. However, workplaces with high demands and insecurity in the workstation led to weak recovery after work^11^, contributing to the deterioration of health in general and to the low productivity of workers.

The socioeconomic status and the nationality of the workers were two variables that influenced how the work psychosocial environment is perceived^23^, and were two important factors for the reduction of social inequalities when there is the intention to intervene in the workplace. Geographic variability and individual cultural specificities were evident as the influence of individual and work factors on the perception of exposure to psychosocial risks, with the most vulnerable groups such as migrants and the non-resident population^27^. The groups of workers displaced from residence show more risks to physical and mental health, especially to hypertension and to the perception of work meaning^27^ ^,^ ^28^. Gender differences were evident in the perception of stress symptoms: women reported greater labor demands and higher levels of stress symptoms when compared with men with analogous functions and tasks^18^.

Older workers showed coping strategies that allowed them to deal more adequately with job demands. The work experience was a mediator factor between age and capability for the work^8^. The professional experience and the physical and cognitive skills also presented themselves as mediators of productivity^j^. Younger employees were the group with higher risk to exposure to psychosocial risk factors, especially of exposure to violent behavior related to work^32^. Age was a risk factor for the performance of tasks with heavier physical loads^15^ and also a resistance and increased resilience factor in the workplace to the psychosocial level^8^ ^,^ ^15^.

Deteriorated work psychosocial environment was associated to weak physical and mental health indicators such as hypertension, MI, stress, low self-esteem, burnout, and health in general^1^ ^,^ ^5^ ^,^ ^7^ ^,^ ^14^ ^,^ ^24^ ^,^ ^27^. A mediocre psychosocial environment was a risk factor for the development of clinical conditions from moderate to severe gravity, causing the absence of the workers in their work, especially for sick leave^29^ or the intention to leave work permanently^18^.

### Synthesis of methodological limitations

The methodology selected for inclusion and exclusion of studies limits the obtained results. It excludes studies with valid data, especially in an area considered to be emerging, as the study of psychosocial factors. This causes relevant factors to the understanding of this subject to be excluded, although little studied.

The selection of studies that use only the COPSOQ measuring instrument of psychosocial factors reduces the studies included in the review, although the methodology allows easier comparison of the identified variables and the nature of the instrument allows covering a greater number of factors.

A quality study on the articles resulting from the application of the methodology was not carried out. This option joined the multidimensional and multidetermined nature of the concept, thus allowing a wider analysis and discussion on the topic.

There is a risk of not including all articles with the defined criteria, using the structured search in databases. To try to mitigate this effect, a third database, Scopus, was included, though it resulted in a full matching of results that did not lead to the inclusion of any study.

There is not enough geographical dispersion. The results may represent exposure to psychosocial risk factors of some geographical areas (e.g., Nordic countries) and with different cultures. One should consider cultural relativity patterns of the populations under study. The same comment applies to the sector of activity. For example, data on the industry area are not reported, and there is evidence in other areas and sectors, such as services or health, in which studies are more plentiful.

Most of the included studies followed a correlational methodological design, and this is not the most appropriate design if we want to understand causality. From the included articles, the more robust experimental designs were little used.

### Implications for practice

An increasing number of organizations is interested in programs promoting the well-being of its employees and management of psychosocial risks, despite the fact that the interventions are commonly focused on a single behavioral factor (e.g., smoking) or on groups of factors (e.g., smoking, diet, exercise). Most programs offer health education, but a small percentage of institutions really changes organizational policies or their own work environment^4^.

This literature review presents important information to be considered in the design of plans to promote health and well-being in the workplace, in particular in the management programs of psychosocial risks. A company can organize itself to promote healthy work environments based on psychosocial risks management, adopting some measures in the following areas:

1. Work schedules – to allow harmonious articulation of the demands and responsibilities of work function along with demands of family life and that of outside of work. This allows workers to better reconcile the work-home interface. Shift work must be ideally fixed. The rotating shifts must be stable and predictive, ranging towards morning, afternoon and evening. The management of time and monitoring of the worker must be especially careful in cases in which the contract of employment predicts “periods of prevention”.

2. Psychological requirements – reduction in psychological requirements of work.

3. Participation/control – to increase the level of control over working hours, holidays, breaks, among others. To allow, as far as possible, workers to participate in decisions related to the workstation and work distribution.

4. Workload – to provide training directed to the handling of loads and correct postures. To ensure that tasks are compatible with the skills, resources and expertise of the worker. To provide breaks and time off on especially arduous tasks, physically or mentally.

5. Work content – to design tasks that are meaningful to workers and encourage them. To provide opportunities for workers to put knowledge into practice. To clarify the importance of the task to the goal of the company, society, among others.

6. Clarity and definition of role – to encourage organizational clarity and transparency, setting jobs, assigned functions, margin of autonomy, responsibilities, among others.

7. Social responsibility – to promote socially responsible environments that promote the social and emotional support and mutual aid between coworkers, the company/organization, and the surrounding society. To promote respect and fair treatment. To eliminate discrimination by gender, age, ethnicity, or those of any other nature.

8. Security – to promote stability and safety in the workplace, the possibility of career development, and access to training and development programs, avoiding the perceptions of ambiguity and instability. To promote lifelong learning and the promotion of employability.

9. Leisure time – to maximize leisure time to restore the physical and mental balance adaptively.

The management of employees’ expectations must consider organizational psychosocial diagnostic processes and the design and implementation of programs of promotion/maintenance of health and well-being. Assessment tools, such as COPSOQ, will early identify psychosocial risk factors and facilitate a more targeted intervention, specific and directed to the said factors concerning the well-being of the individual and the best ability to work. The results obtained with an intervention in the workplace may not be always positive or desired. The negative effect of interventions^3^ may arise, in which the optimism about organizational interventions acts as a barrier to development and elaboration of differentiated forms of intervention^2^.

## CONCLUSIONS

We presented the relevance and surplus value of the use of a multidimensional instrument, when there is the intention to intervene in organizational context. The nature of the factors under study – psychosocial risk factors – and the multiplicity of individual and labor factors may influence the relationship. Appealing to validated instruments with the possibility to adapt to the reality and context of each company, such as the COPSOQ, is a surplus value. The resulting data of this evaluation instrument of psychosocial risks can be compared with international reference values, which facilitates signaling deviations related to reference points in the population, facilitating the intervention. Therefore, what matters is to study and understand as many variables as possible to support an “emerging” field of study and the practical intervention.

We observed interaction between individual and work-related factors, and psychosocial risk factors. The exposure of workers to poor psychosocial environments influences various levels, from the physical and mental health of employees to the general work environment of institutions and the quality of leisure and rest time. It is necessary to understand the extent of these factors in population groups little studied, such as the industry, and to use more robust experimental plans.

## Footnotes

a European Agency for Safety and Health at Work. Research on the changing nature of work. Luxembourg: Office for Official Publications of the European Communities; 2002.

b European Agency for Safety and Health at Work. Expert forecast on the emerging psychosocial risks related to occupational safety and health. Luxembourg: Office for Official Publications of the European Communities; 2007. (European Risk Observatory Report, nº 50).

c US Department of Health and Human Sciences, Centers for Disease Control and Prevention, National Institute for Occupational Safety and Health. The changing organization of work and the safety and health of working people: knowledge gaps and research directions. Cincinnati: NIOSH; 2002.

d International Labour Organization. Psychosocial factors at work: recognition and control. Geneva: International Labour Office; 1986. (Occupational and Health Series, 56).

e See the instrument validation manuals for different languages/countries, e.g., Silva CF. Copenhagen Psychosocial Questionnaire COPSOQ: Portugal e países africanos de língua oficial portuguesa. Lisboa: Fundação para a Ciência e a Tecnologia; 2000. Instituto Sindical de Trabajo y Salud, Environment). Manual del método CoPsoQ istas 21 para la evaluación y prevención de los riesgos psicosociales para empresas con 25 o más trabajadores y trabajadoras. Versión 1.5. Barcelona: Centro de Referencia de Organización del Trabajo y Salud; 2010.

f PRISMA Transparent Reporting of Systematic Reviews and Meta-Analyses. Ottawa: The PRISMA Statement; 2014 [cited 2014 Dec 30]. Available from: http://www.prisma-statement.org/

g European Commission Directorate-General for Employment, Industrial Relations and Social Affairs. Guidance on work related stress: spice of life or kiss of death? Luxembourg: Office for Official Publications of the European Communities; 2000.

h International Labour Organization. List of occupational diseases: revised 2010. Geneva; 2010.

i International Labour Organization. Mental health in the workplace. Geneva; 2000.

j Hunkler c. Age and productivity: speech at the European Summit on Innovation for Active and Healthy Ageing; 2015 Mar 9-10; Brussels, Belgium. Munich: Max Planck Institute for Social Law and Social Policy; 2015 [cited 2015 Apr 14]. Available from: http://ec.europa.eu/research/innovation-union/pdf/active-healthy-ageing/hunkler.pdf#view=fit&pagemode=none/
